# Supplementary material for: Short-Chain Fatty Acids Ameliorate Depressive-like Behaviors of High Fructose-Fed Mice by Rescuing Hippocampal Neurogenesis Decline and Blood–Brain Barrier Damage
Source: Nutrients. 2022 Apr 29;14(9):1882. doi: 10.3390/nu14091882 (PMC9105414; doi:10.3390/nu14091882)
Supplement: Supplementary file 1 [file nutrients-14-01882-s001.zip › nutrients-1679541-supplementary.pdf]

nutrients

Supplementary Materials

SCFAs Enhance Stress Resilience of Mice Fed by High-Fructose Diet through Improving Hippocampal Neurogenesis Decline and Blood–Brain Barrier Damage

Chuan-Feng Tang [1], Cong-Ying Wang [1], Jun-Han Wang [1], Qiao-Na Wang [1], Shen-Jie Li [2], Han-Ou Wang[2], Feng Zhou [2,*] and Jian-Mei Li [1,*]

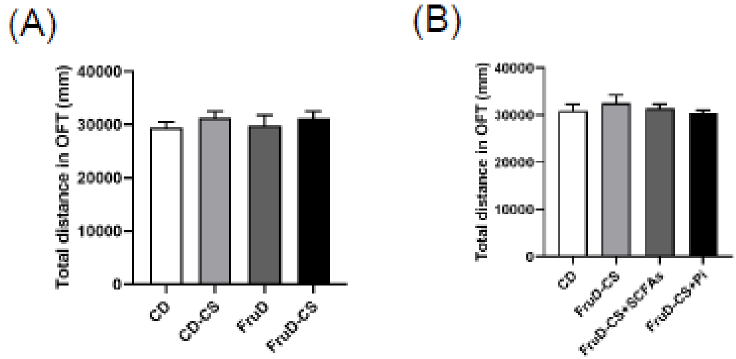

Figure S1. FruD, chronic stress or SCFAs supplementation failed to change the spontaneous activity of mice in OFT. Total distance of mice in OFT (**A,B**).
